# Exploring Thailand's mortality transition with the aid of life tables

**DOI:** 10.1111/j.1467-8373.2010.01436.x

**Published:** 2011-04

**Authors:** Gordon A Carmichael

**Affiliations:** National Centre for Epidemiology and Population Health, Australian National UniversityCanberra, ACT 0200, Australia. Email: gordon.carmichael@anu.edu.au

**Keywords:** HIV/AIDS, infant mortality, life expectancy, mortality transition, road deaths, Thailand

## Abstract

The project Thai Health-Risk Transition: A National Cohort Study seeks to better understand the health implications of modernisation and globalisation forces impacting on Thailand. As part of its ‘look-back’ component this paper seeks, using available life tables, to document the country's post-war mortality transition. The onset of transition through mass campaigns of the late 1940s and 1950s is first discussed before attention turns to the life tables. They are predictably far from flawless, but careful analysis does permit trends that have seen around 30 years added to life expectancy to be traced, and age patterns of improved survivorship and their relation to initiatives to improve health to be examined. The broad benefits generated by mass campaigns, ongoing improvements in infant and early childhood mortality, and a phased impact of the expansion of primary health care in rural areas on adult survival prospects after the mid-1970s are demonstrated. The paper also investigates the consequences for mortality of a motorcycle-focused rapid increase in road fatalities in the late 1980s and early 1990s and the HIV/AIDS epidemic that developed after 1984.

## Introduction

**Figure 1:**
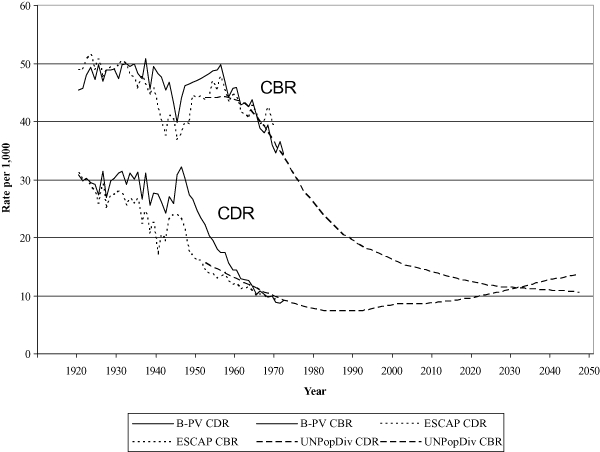
Thailand: Crude death rate (CDR) and crude birth rate (CBR) estimates 1920–2047. B-PV, estimates of Bourgeois-Pichat (1959) 1920–1955 updated to 1972 by Vallin (1976).

Demographic transitions are typically illustrated by plotting together trends in crude birth and death rates. In the classic scenario both rates are initially similar and high, indicating slow population growth; the crude death rate declines, ushering in rapid growth; decline in the crude birth rate follows, slowing growth again; then a new equilibrium is established with both rates low. Estimates for Thailand available from 1920 do not show pre-transitional equilibrium (Fig. 1). Moreover, it has been argued (Carmichael, 2008) that the *dis*equilibrium then apparent reflects not just mortality decline but fertility *in*crease, as development of a labour-intensive wet-rice economy after 1850 combined with cessation of indigenous warfare and the demise of corvée labour and slavery to create, for Thailand, a historically unique domestic environment – sedate, settled, optimistic and hence pronatalist.

Fascinating though the possible pre-1920 course of birth and death rates in Thailand is, however, the bulk of the country's demographic transition has occurred post-World War II. The focus here is on insights into its mortality component provided by life tables available from 1937 onwards. Several life table functions are examined as the improvement in life expectancy is unpacked, but special attention is paid to the _n_d_x_, or life table deaths (see later technical discussion), columns of abridged life tables used, where changes strikingly identify ages at which deaths were mainly prevented over successive periods.

Thailand is not alone in Southeast Asia in having experienced substantial mortality decline. Caldwell (2006) traces the tortuous path of socio-economic, cultural and scientific advance that underpinned mortality transition in Europe, highlighting as key the development of microscopes which finally facilitated the understanding of infectious disease transmission. By contrast, developing countries after World War II had available (from Europe) an array of new drugs and insecticides and older vaccines that enabled several of them, aided by new global funding agencies, to rapidly lower mortality without significant increases in living standards (Bloom and Williamson, 1998; Caldwell, 2006). This contrast is very effectively illustrated for Asia in Rosling's (2010) animation of the changing relationship over time between life expectancy and per capita income for 200 countries. For European nations the two measures essentially increased in tandem; for most Asian ones there were initially substantial rises in life expectancy after World War II, with major movements in income per capita following later.

Mortality fell fastest in East Asia (Bloom and Williamson, 1998; Zhao and Kinfu, 2005), while Sri Lanka enjoyed a successful anti-malaria campaign (Pinikahana and Dixon, 1993), but Thailand was among the more proactive Southeast Asian countries in exploiting the opportunity. By 1965–1970 it ranked regionally in life expectancy behind only Singapore and Brunei, level with Malaysia, a little ahead of the Philippines, and 8–13 years ahead of Myanmar, Vietnam, Indonesia, Laos and Cambodia. Twenty years later longevity had improved in all these countries, but Indonesia had left Laos and Cambodia trailing (the latter having seen life expectancy plummet to 31 years under Pol Pot before recovering) and surpassed Myanmar, Vietnam had also overtaken Myanmar (and was ahead of Indonesia), and the Philippines had progressed more slowly than Thailand and Malaysia. Thailand, however, was about to experience an explosion of road deaths and an AIDS epidemic, so that by millennium's end male and overall life expectancy had fallen again, Malaysia and Vietnam had forged ahead, the Philippines had caught up, and Indonesia was closing fast. Yet the success of Thailand's response to AIDS, which averted over 5 million infections and kept to roughly 2% an adult infection level that might have reached 10–15% (East-West Center, 2002), means that this setback will probably prove to be temporary. Hugo (2003: fig. 3.1) rates Thailand behind only Singapore within Southeast Asia in progress through the full demographic transition. Its problem has been rising young adult mortality, but on the key transition indicator of infant mortality it currently ranks, at 7 deaths per 1000 live births, behind only Singapore (3) and Brunei (6), ahead of Malaysia (9) and well ahead of Vietnam (20), the Philippines (23), Indonesia (27), Laos (50), Myanmar (61), and Cambodia (62). The latter countries clearly have some way to go to complete mortality transitions, but all Southeast Asian nations except Singapore have distinct potential to further lower mortality if current life expectancies in developed countries, including Japan, Hong Kong, South Korea and Taiwan, are any guide.

Data for constructing Thai life tables are far from ideal. Birth and death registration data exist from 1920 (Rungpitarangsi, 1974; Rachapaetayakom, 1976; Prasartkul and Vapattanawong, 2006), but significant under-registration (Bourgeois-Pichat, 1959; ESCAP Secretariat, 1976; Tangcharoensathien *et al*., 2006) and age-selective under-enumeration in census data, especially at ages zero to four (Caldwell, 1967; ESCAP Secretariat, 1976; Luther *et al*., 1986), mean that life tables necessarily incorporate significant estimation and assumption or are survey based.

Bourgeois-Pichat (1959) estimated under-registration in deriving annual estimates of Thai crude birth and death rates for 1920–1955 (solid lines in Figure 1, updated to 1972 by Vallin, 1976), but the issue was seriously confronted only when the National Statistical Office (NSO) initiated decennial Surveys of Population Change (SPCs) in 1964–1967. These have sought through multi-round, kingdom-wide household surveys to enumerate birth and death events, then through matching with vital registration data (mid-1960s to mid-1980s) or inquiring whether they had been registered (1995–1996 and 2005–2006), to assess the degree to which vital registration captured those events. Significantly, SPCs also became a basis for constructing life tables without registration and census data.

The first SPC estimated that in the mid-1960s only 65% and 60% of male and female deaths, respectively, were registered. Registration was especially incomplete for infant deaths (50% and 47% registered) (Lumbiganon *et al*., 1990) and was poorer for female deaths at all ages (NSO, 1969). Later surveys suggested a deterioration in male death registration by the mid-1970s (58%) but little change for females (61%), followed by big improvements to the mid-1980s (75% and 77%, respectively) and mid-1990s (95% for both sexes) (Prasartkul and Vapattanawong, 2006). Because they derive from self-reported data, however, Prasartkul and Vapattanawong doubt the accuracy of mid-1990s figures. So do Hill *et al*. (2007), who, using two methods of indirect adjustment for underreporting of deaths, claim that the completeness of death registration actually *deteriorated*, from 78–85% during 1980–1990 to 64–72% during 1990–2000.

The 2005–2006 SPC replicated the 1995–1996 approach, except that interviewers asked to sight death certificates to verify self-reported registrations. Estimates of 94.8% and 95.7% of male and female deaths registered resulted (NSO, 2007). Fieldwork conducted in late 2007, however, disclosed appreciable scepticism among Thai academic demographers about the capacity of SPCs to accurately gauge the completeness of death registration. SPCs recruited baseline samples of households which were then revisited four times at three-month intervals and asked about intervening vital events. This placed a premium on recruiting residentially stable households, whereas failure to register deaths could be associated with transience. Moreover, knowledge that one's household would be revisited encouraged registration of vital events. In Thailand this is a two-step process. Depending on the location and circumstances of death, relatives receive interim documentation from a medical facility, village headman, local administrative officer or policeman (Step 1) to take to a registration office (Step 2). But if a Buddhist monk accepts the interim documentation to initiate funeral arrangements, then Step 2 might not be taken.

Under-enumeration of young children and under-registration of deaths among them are major impediments to accurately quantifying mortality trends in Thailand. That said, from 1960, census and survey data permitting the application of robust indirect methods for estimating infant and early childhood mortality are available. Limitations in the data from which Thai life tables have been constructed nonetheless need to be appreciated. This includes SPC-based tables, which feature idiosyncrasies of detail that betray little effort to identify and smooth anomalies. In keeping with the demographer's tradition of making the best of available data, however, the careful analysis that follows does help elucidate major phases of Thailand's mortality transition.

## Onset of the post-war mortality transition

As the twentieth century unfolded, new public health initiatives were undertaken in Thailand. Primary health care dates from 1909, when the Provincial Sanitary Organization began establishing health centres and clinics in small towns and rural communities. By 1936 they operated at 136 localities (McGuire, 2010). A 1911–1912 Bangkok smallpox epidemic led to vaccination being made mandatory upon ministerial order there, then more generally across the country. Before World War II the scourge of smallpox seemed to be waning, an annual average of just 279 cases being recorded during 1920–1943. However, 925 cases in 1944 heralded severe epidemics in 1945 and 1946 that claimed 15 000 lives from 63 000 cases (Fenner *et al*., 1988). Other initiatives included a Sanitary Campaign launched in 1917 to tackle hookworm (Varavarn, 2000), and a 1932 expansion of rural health services that saw several provincial hospitals built in the North and Northeast, and the first *tambon*-level^1^ health centres established (Porapakkham, 1986). The Sanitary Campaign took health information to 44 provinces, deployed nine field units of ‘sanitary inspectors’ (Varavarn, 2000: 190) (including one serving the riparian and floating population of the Chao Phraya river), and erected thousands of latrines. After 1929 it was integrated into the broader public health effort, but in 1930 Varavarn wrote:

It cannot be sufficiently emphasized that the relief and control of hookworm disease … has been more than an end in itself. [The Sanitary Campaign] has lent itself well for the purpose of demonstrating the course, the mode of transmission, the cure and the means of prevention of a large and important group of diseases (Varavarn, 2000: 193).

The major transition to low mortality occurred, however, after World War II. Hirschman (1994: 396) writes that there was ‘probably little progress in mortality’ in Southeast Asia from the early 1930s to the mid-1940s. ‘(A) unique period of extraordinarily rapid population growth’ then followed that was ‘primarily a result of record declines in mortality’. Less densely settled countries like Thailand were at the forefront.

Until the 1930s when sulfa drugs were introduced, the ability of medicine to cure disease was limited to first aid and nursing care. On the heels of sulfa drugs came penicillin and other antibiotics in the late 1940s and 1950s. … The other postwar development was … massive public health campaigns. DDT spraying in the late 1940s and 1950s helped reduce malaria. There were also large-scale programmes to inoculate school children against most major childhood endemic diseases (Hirschman, 1994: 402).

In Thailand, eradication of infectious diseases like yaws, plague and smallpox was pursued, malaria was greatly reduced by DDT spraying, an effort was made to contain tuberculosis, and production of medical graduates and provision of rural health facilities accelerated. Provincial hospitals increased from 14 in 1942 to 20 in 1950 and 72 in 1956 (Porapakkham, 1986; Muscat, 1990); rural health centres increased from 490 in 1946 to 1363 in 1959 (McGuire, 2003a,b;). Yaws succumbed to the ‘magic bullet’, penicillin, a million cases being cured by mobile teams and the disease virtually eliminated during the 1950s (UNICEF, 1995). Plague reached Bangkok from India in 1904, a 1917 epidemic producing over 500 deaths (Elbel and Thaineua, 1957). After cases fell to zero during 1935–1937 it emerged again through the war and early post-war years, but the creation of laboratories for detection and control in three endemic plague areas in 1952 saw it disappear by mid-decade (Velimirovic, 1972). Smallpox, for centuries a source of epidemic mortality (Terweil, 1987), was targeted by an intensive vaccination campaign. This seemed to have succeeded by the mid-1950s, but following a resurgence of 1500 cases in 1959 the disease's eradication was finally declared in 1962 (Fenner *et al*., 1988).

The public health hazard malaria constituted before the 1950s was summarised by Dr Ira Ayer, a Department of Public Health adviser writing in 1926:

Year in and year out malaria justifies its characterization … as the greatest single destroyer of the human race. When one has seen a fever-stricken village with more than half the population sick, with perhaps a death in every household, it makes an impression not easily forgotten. Between 40 000 and 50 000 deaths from malaria have been reported in a single year … quinine is distributed in increasing quantity but, owing to its extent and complexity, the malarial problem in Siam is yet far from practical solution (cited by Varavarn, 2000: 224).

Eradication of malaria was not initially the target, but it became the target briefly after the success of initiatives during the 1950s. Before 1950 malaria infected 4–5 million Thais and caused 40 000 deaths annually (Thurman, 1962). However, a WHO/UNICEF pilot project in Northern Thailand in 1949–1950 demonstrated the efficacy of residual DDT spraying of houses for malaria control. Thailand sought US aid to extend the programme, and with cooperation from Buddhist monks to overcome the religious ban on killing animals (Thurman, 1962), the homes of 14 million people in malarial areas were treated between 1951 and 1958 (Muscat, 1990). The malaria death rate fell from 201.5 per 100 000 in 1949 to 24.5 in 1961 (Caldwell, 1967; Ministry of Public Health, n.d.), and malaria had declined from being the leading cause of death to fifth position by 1958 and to eighth position by 1968 (McGuire, 2003a,b;).

The incentive this success provided to attempt eradication was thwarted by the development and spread of drug-resistant parasites and insecticide-resistant mosquito vectors, leading to the reinstatement of ‘control’ as the goal from the early 1970s. This backtracking was part of a wider international recognition that ‘eradication’ was an overly optimistic objective in respect of several infectious diseases (Yekutiel, 1981; Williams, 1988; Stern and Markel, 2004). Malaria remains a public health issue along the Thailand–Myanmar and Thailand–Cambodia borders (Thimasarn *et al*., 1995; Konchom *et al*., 2005; Ministry of Public Health, n.d.), but by 2003 the official death rate was just 0.3 per 100 000 (Ministry of Public Health, 2005: fig. 5.7).

Tuberculosis, second only to malaria as a cause of death (Prakalapakorn, 2000), was another post-war public health target, and in 1949 a chest clinic opened in central Bangkok. In 1951 it became one of four Asian WHO/UNICEF-backed TB training and demonstration centres. Mass BCG vaccination of school children occurred during 1953–1956, followed by a consolidation phase with mobile vaccination teams visiting provinces every three to four years (Sriyabhaya *et al*., 1993). Chemotherapy then brought a decision to concentrate on expanding ambulatory treatment services rather than building more TB hospitals. Therefore, from 1959 regional and zonal TB centres were created nationwide for case detection and service provision (Sriyabhaya *et al*., 1993; Prakalapakorn, 2000; Palwatwichai, 2001).

## A life table perspective on the mortality transition

The ensuing analysis uses life tables from three sources to trace improvements in male and female life expectancy in Thailand and to identify age groups where mortality reductions were concentrated over different periods. In assembling them the aim was to obtain a series from as few reputable sources as possible, to minimise methodological inconsistency across the 73-year period covered (1937–2010).

Life tables were obtained for 1937, 1947, 1960 and 1970 from Rungpitarangsi (1974), for 1964–1967, 1974–1976, 1985–1986, 1995–1996 and 2005–2006 from SPCs (NSO, 1969, 1978, 1987, 1997, 2007), and for 1980–1985, 1985–1990, 1990–1995, 1995–2000, 2000–2005 and 2005–2010 from the United Nations (UN), whose 2008 revision of *World Population Prospects* produced two sets of tables – including and excluding AIDS mortality. Period life tables^2^ at five-year intervals 1900–2000 were also available from Prasartkul and Rakchanyaban (2002). These were generated as an intermediate step in producing cohort life tables^3^ by first estimating life expectancies at birth by fitting logistic curves to expectancies, by sex, from the Rungpitarangsi and SPC tables, then obtaining _n_q_x_-values (age-specific probabilities of dying) from Coale and Demeny's (1983) ‘West’ model life tables.^4^ They were used in a support role, described below. In respect of life expectancy, although not necessarily some of their detail, SPC life tables overlap Rungpitarangsi's neatly, while 1985–1986 SPC tables blend reasonably smoothly with those from the UN.

Rungpitarangsi (1974) generated her life tables using a method developed by William Brass (described in Brass, 1975) for adjusting age-sex-specific death rates based on defective registration and census data. She acknowledges, however, using (p. 65) ‘rough estimates’ at ages 0, 1–4, and 5–9 years, where Brass's procedure was inappropriate, and for 1937 and 1947 her age 0 (infant mortality) levels seem *very* rough estimates.

Varavarn (2000: 217) claims that for the decade ended March 1930, annual infant mortality rates for Bangkok Registration Area averaged 234 deaths per 1000 live births. He places the national figure (p. 228) at ‘approximately one third’. Rungpitarangsi (1974: 69), possibly drawing on this same evidence, likewise proclaims infant mortality in the mid-1920s ‘as high as 300 per 1000 live births’, but the ‘rough estimates’ in her 1937 life tables are far lower – 129.2 per 1000 male and 109.5 per 1000 female births. If the 1920s estimates are ‘ballpark’ correct, these numbers imply hugely reduced infant mortality during 1930–1937, the basis for which Rungpitarangsi never addresses. Judged against indirect estimates McGuire (2003a,b;) assembles and ‘West’ model values adopted by Prasartkul and Rakchanyaban (2002), Rungpitarangsi's infant mortality assumptions for 1960 and 1970 are plausible, but those for 1937 and 1947 are extremely dubious. Investigation did not disclose a similar problem at ages one to four.

Accordingly, and with Riley (2005: 52) having commented that in terms of international comparative perspectives the expectations of life at birth in Rungpitarangsi's 1937 and 1947 life tables are ‘surprisingly high for a country at the beginning of a health transition’, it was decided to regenerate these life tables, replacing their infant mortality levels with values interpolated from Prasartkul and Rakchanyaban's (2002) ‘West’ model-based tables. Male and female infant mortality rates for 1937 rose from 129.2 to 195.7 and from 109.5 to 171.6 deaths per 1000 live births, respectively; those for 1947 rose from 121.8 to 178.3 and from 102.7 to 147.5. Life expectancies at birth for both sexes at both dates consequently fell by around three years.

Difficulties in estimating mortality are not confined to the youngest ages, as can be appreciated from Rungpitarangsi's (1974) account of her estimation procedures and from noting some curious irregularities in SPC life tables (see below). It is thus inconceivable that Thai life tables are error-free at older ages either, so interpretation of trends they reveal requires caution.

### Broad trends in survivorship

**Figure 2:**
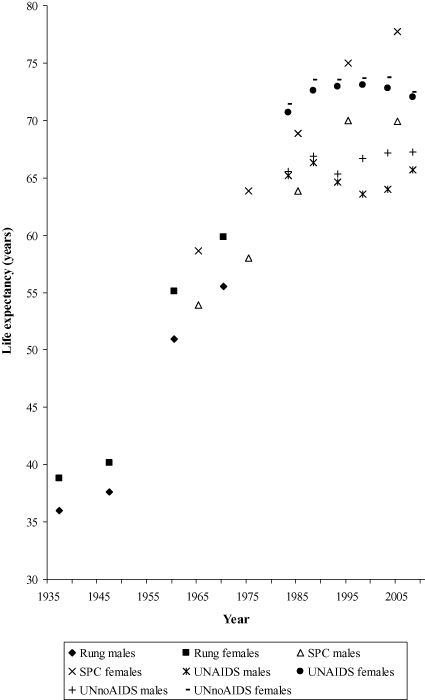
Thailand: Expectations of life at birth by sex 1937–2010. Rung, Rungpitarangsi (1974); SPC, Survey of Population Change (Thai National Statistical Office); UNAIDS, UNnoAIDS, United Nations, *World Population Prospects 2008*, estimates including and excluding AIDS mortality.

Figure 2 plots male and female expectations of life at birth from the Rungpitarangsi, SPC and UN life tables. Data points follow quite smooth upward trends until the late 1980s, after which complications associated with rising road accident mortality and HIV/AIDS intrude. Female life expectancy, however, consistently exceeds that for males.

The adjusted 1937 life expectancies for males (36.0 years) and females (38.8 years) in Figure 2 rise modestly to 37.6 and 40.1 years, respectively, by 1947. Thereafter there are huge increases to 50.9 and 55.1 years in 1960 – increments of 13.3 and 15.0 years, respectively. The decade and a half post-war was really a period of massive improvement in survival prospects in Thailand, largely divorced from major economic development (Ayal, 1961). Rungpitarangsi's tables then show male and female life expectancy rising a further 4.6 and 4.7 years to 55.5 and 59.8 years, respectively, between 1960 and 1970. Comfortingly, life expectancies from the 1964–1967 SPC tables lie neatly between her 1960 and 1970 figures. Those from the 1974–1976 and 1985–1986 SPCs (males 58.0 and 63.8 years; females 63.8 and 68.9 years) then continue the upward trends, the mid-1980s figures indicating 15-year increments of 8.3 and 9.1 years for males and females compared with the 1970 Rungpitarangsi figures.

Beyond the mid-1980s Figure 2 becomes more confused. There is controversy over more recent trends in Thai life expectancy, centred on the doubts over 1995–1996 and 2005–2006 SPC estimates that registration of deaths was 95–96% complete, and on the effect of HIV/AIDS. SPC female life expectancy trends relentlessly up to 77.7 years in 2005–2006, but this figure seems inflated, not least by implausibly low infant and child mortality compared with that in the equivalent male life table.

The UN estimates that incorporate AIDS mortality (‘all-causes’ estimates) show higher female life expectancy for 1980–1985 than the 1985–1986 SPC shows (70.7 years compared with 68.9 years), but rise more sedately thereafter to a peak of 73.1 years in 1995–2000, below the 1995–1996 SPC level and well below that for 2005–2006. There is a distinct flattening of upward momentum after 1985–1990, then a decline beyond 1995–2000. ‘No-AIDS’ life expectancy similarly flattens off and also declines beyond 2000–2005, but both declines are probably spurious. An obvious question to ask is: ‘What non-AIDS causes of death were responsible for the latter decline?’ The answer is probably ‘none’. ‘No-AIDS’ mortality is estimated *as a residual*; all-causes mortality and mortality from AIDS are separately modelled, then the latter is subtracted from the former. UN advice is that Thai all-causes mortality for 2000–2005 and 2005–2010 was modelled *without* factoring in rapidly expanding access to antiretroviral therapy (ART), but estimation of AIDS mortality *did* factor this in (a rise from zero coverage in 2000 to an estimated 34% in 2005 and 61% in 2007 following the promulgation of a universal coverage policy in October 2003 (Steinbrook, 2007; UNAIDS, 2008). Two things follow from this curious disclosure. First, the post-1995–2000 decline in female all-causes life expectancy is dubious because it fails to reflect reduced AIDS mortality as a result of ART. Second, because of their residual character, ‘no-AIDS’ mortality estimates for 2000–2005 and 2005–2010 also incorporate that component of the AIDS mortality in the all-causes estimates that was actually eliminated by ART; only the AIDS mortality that remained after taking ART into account was subtracted.

For males the SPC data (Fig. 2) show a continued steep upward trend in life expectancy to 1995–1996, followed by a slight decline to 2005–2006 at 69.9 years. UN data paint a different picture. As with females, all-causes mortality estimates show slightly higher life expectancy for 1980–1985 than does the 1985–1986 SPC. Both all-causes (‘AIDS’) and ‘no-AIDS’ life expectancy then rise to 1985–1990 and fall to 1990–1995 in tandem before following distinct AIDS-related paths (although also impacted beyond 1995–2000 by the ART issue). The mortality impact of AIDS was much more severe for males, reflecting the epidemic's initially residing in the sex industry. But the tandem decline between 1985–1990 and 1990–1995 is also of interest. It implies that AIDS was *not* the main cause.

The epidemic was well established by the early 1990s, but death in the absence of ART seems not to typically have occurred in Thailand until eight years post-infection (Nelson *et al*., 2007; Rangsin *et al*., 2007). Something else impacted young adults – a huge rise in traffic deaths (mainly involving motorcycles) that Sintuvanich (1997) and Suriyawongpaisal and Kanchanasut (2003) associate with industrialisation. Faculty of Engineering, Prince of Songkla University (FEPSU) (2007: table 1.1) data show an eightfold rise in road fatalities, from 2104 to 16 727, between 1987 and 1995. Numbers then dropped with the phasing in during 1995 of a law prescribing helmet use by motorcyclists and their passengers (Ichikawa *et al*., 2003; Pitaktong *et al*., 2004; Kanitpong *et al*., 2008) but still averaged 13 000 annually during 1996–2005. The trend accompanied a rapid increase in motor vehicle use beyond Bangkok, which accounted for 36% of traffic deaths in 1987 but less than 8% by 1995. Over 60% of victims were aged 15–39, and 80% were male (Ekachampaka and Wattanamano, 2008: table 5.25 and fig. 5.43), although more traffic deaths will also have helped slow the rise in female life expectancy. Motorcycles quintupled from 2 to 10 million during 1984–1995 (Tangpaisalkit, 2004), and while by the late 1990s they were a third of vehicles involved in road accidents (FEPSU, 2007: table 1.2), injury surveillance data show riders and pillions accounting during 1997–1999 for a staggering three-quarters of road fatalities (Ekachampaka and Wattanamano, 2008: table 5.27).

Rumakom *et al*. (2003) and Hill *et al*. (2007) argue that AIDS ‘interrupted’ epidemiological transition in Thailand, although Vallin (2007: 386) declares this interpretation ‘quite inappropriate’ given the rapid, effective response to the crisis (Porapakkham *et al*., 1995; East-West Center, 2002; United Nations Development Programme, 2004; Phoolchareon, 2006). Rumakom *et al*. note a re-emergence of infectious diseases, especially tuberculosis and pneumonia, and that AIDS was often the underlying cause of deaths attributed to them. Mortality increases were concentrated among males aged 20–44. Hill *et al*., using life tables based on their own rather than SPC completeness of death registration estimates, report (2007: 381) ‘substantial increases in adult mortality over a very short period’. They were concentrated at ages 20–39, and although offset by reduced ‘under-five’ mortality yielded a decline of four years (from 66.6 to 62.6 years) in male life expectancy between 1980–1990 and 1990–2000, and only a marginal increase for females (from 73.0 to 73.2 years).

Hill *et al*. (2007) briefly acknowledge the likelihood that increased adult mortality also derived from more road deaths, although they probably merited less incidental mention. The gap of just over six years between female and male life expectancy they obtain for the 1980s is wider than that of five years yielded by the 1985–1986 SPC life tables but similar to the average of the UN ‘all-causes’ gaps for 1980–1985 and 1985–1990 (5.9 years). That of over 10 years they derive for the 1990s compares with a peak UN gap for 1995–2000 of 9.5 years, while their four-year decline in male life expectancy compares with a UN decline from 66.3 to 63.6 years between 1985–1990 and 1995–2000. Following the rapid response to the AIDS epidemic and the advent of ART, young adult male mortality clearly fell again beyond 2000, and once adjustments for ART are built into the next revision of UN estimates, all-causes male life expectancy for 2005–2010 should rise from 65.7 years in the 2008 estimates, and the gender gap should again be around six years.

Historically, data plotted in Figure 2, including the recalculated 1937 and 1947 estimates substituting more plausible infant mortality levels, suggest that over the six decades from 1947, life expectancy at birth for Thai males improved by around 28 years and that for Thai females by about 32 years. The bulk of these increments were achieved in the first four of those decades. Therein lies the broad outline of Thailand's mortality transition.

### Mortality trends 1937–1970

**Figure 3:**
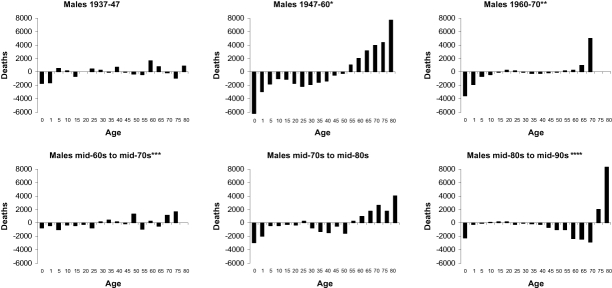
Changes in life table deaths between successive male life tables, 1937 to mid-1990s (radix = 100 000). *Plot for age group 0 = −6140; **Terminal age group 70 and over; ***Terminal age group 75 and over; ****Plot for age group 80+ = 10 997.

**Figure 4:**
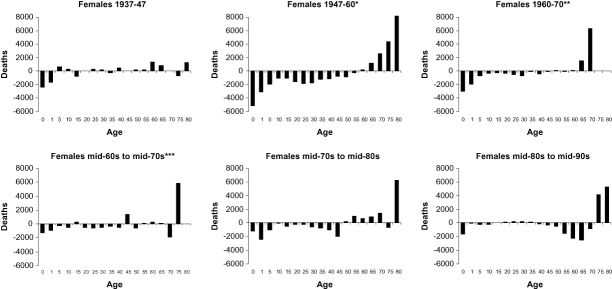
Changes in life table deaths between successive female life tables, 1937 to mid-1990s (radix = 100 000). *Plot for age group 80+ = 13 425; **Terminal age group 70 and over; ***Terminal age group 75 and over.

Attention now turns to comparing successive pairs of life tables to pinpoint population segments that benefited as Thai life expectancy rose. In a novel approach, this is facilitated primarily by comparing _n_d_x_ (age-specific deaths) columns of life tables, the idea being to focus on how age distributions of the 100 000 deaths in any life tables^5^ changed from one life table to the next. At what ages were numbers (effectively proportions) of total deaths reduced, and where did compensating increments in a zero-sum situation occur (because death can be deferred but never avoided)? Graphs in Figures 3 and 4 traverse the 1937–1970 Rungpitarangsi life tables (recalculated for 1937 and 1947), and the SPC series between the mid-1960s and mid-1990s. Bars below the horizontal axis indicate fewer deaths at the later date; those above it indicate more deaths. Sums of below and above bars are identical. Age groups labelled ‘0’ and ‘1’ refer to ages zero and one to four. Others are five-year groups commencing at the age indicated (e.g. ‘5’ means ‘five to nine’), except that the oldest group, ‘x’, is age group ‘x or older’.

The upper-left graphs in Figures 3 and 4 (1937–1947) show no pattern to life table deaths changes, which are mostly small. Much more impressive are the 1947–1960 graphs, portraying major transfers of mortality from ages under to ages over 55 for males and 60 for females, with reduction peaks in infancy and at ages 25–29. These patterns are indicative of the broad benefits reaped from advances in control of malaria and other infectious diseases during the 1950s. While mass campaigns later fell into some disrepute, there is no denying their major public health benefit to Thailand at this time. The large reductions in infant deaths shown reflect the higher infant mortality levels assumed in regenerating Rungpitarangsi's 1947 life tables. They are subject to error, but the pattern of improvement can safely be said to be far closer to reality than had her 1947 tables been accepted uncritically.

**Figure 5:**
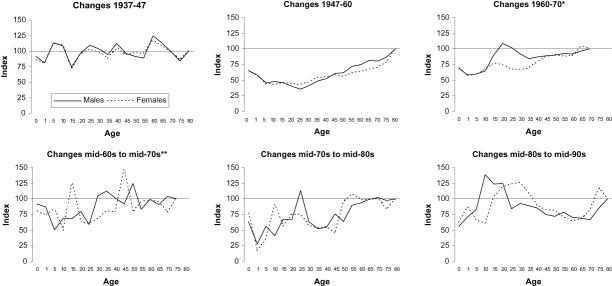
Changes in age-specific probabilities of dying between successive life tables by sex, 1937 to mid-1990s. *Terminal age group 70 and over; **Terminal age group 75 and over.

The upper right graphs in Figures 3 and 4 indicate that during the 1960s mortality gains were heavily concentrated in infancy, especially, and childhood, with offsetting increments in deaths concentrated at ages 65 and older. Improved female mortality at reproductive ages is also suggested and is highlighted in Figure 5, which for periods covered in Figures 3 and 4 plots end-date age–sex-specific probabilities of dying against start-date index values of 100. The lack of pattern to 1937–1947 changes is confirmed. The graph for 1947–1960 then shows probabilities falling at all ages, including older ones, but most sharply in later childhood, adolescence and early adulthood. Older age improvements are not apparent in Figures 3 and 4 because those at younger ages left many more survivors at older ages. Thus, despite declining *probabilities* of dying at those ages, *numbers* of life table deaths rose. An interesting feature of Figure 5, though, is the separation of male and female trend lines across reproductive ages for 1960–1970. This points to improved maternal mortality, and Ministry of Public Health (2005: fig. 5.1) data show the maternal mortality ratio falling almost 40% between 1962 and 1970.

The 1960s were an experimental phase in upgrading rural primary health care in Thailand. Krongkaew (1982) and Nitayarumphong (1990) describe pilot projects begun in 1964 in Pitsanuloke and 1968 in Saraphi that aimed to provide ‘available, accessible, affordable and acceptable’ services (Ministry of Public Health cited by Krongkaew, 1982: 92). The former was ‘only a partial success’, unable to maintain regular supervision of volunteer staff or a system of patient referral (Krongkaew, 1982: 92). The Saraphi Project pioneered the use of village health volunteers (VHVs) and village health communicators (VHCs), who, together with the deployment of *wechakorn* paraphysicians and the upskilling of traditional birth attendants, were central to the more ambitious Lampang Project, launched with USAID in 1974 (with respective training periods of two weeks, two days, a year and two weeks). The late 1950s and 1960s also brought an effort to improve water and sanitation through the Village Health and Sanitation Program, as gastrointestinal diseases were targeted (McGuire, 2003b). However, the programme of well digging, outhouse construction, and village sanitation committee creation was ‘modest’ and ‘limited by lack of coordination … and problems maintaining the hand pumps’ (McGuire, 2003b: 19). Almost no Thai households in 1960 had ‘safe’ drinking water or ‘sanitary’ latrines, and proportions reached just 8.5% (water) and 20.1% (latrines) by 1970 (McGuire, 2003a: table 5.5).

### Mortality trends mid-1960s to mid-1980s

The lower graphs in Figures 3 and 4 plot changes in SPC life table deaths between the mid-1960s and mid-1990s. Left graphs overlap upper-right graphs but show less improvement in infant and early childhood mortality. This does not mean improvement was concentrated in the earlier 1960s, indirect estimates (McGuire, 2010: table A1) pointing emphatically to decade-long improvement. Inspection of relevant SPC life tables suggests flaws in their infant mortality estimates. Hill *et al*.'s (1999) reconciliation of indirect estimates puts Thai infant mortality at 86 per 1000 live births in 1965 and 62 in 1975. By comparison, means of male and female infant mortality rates in 1964–1967 and 1974–1975 SPC life tables are too low (78.3) then too high (68.0), more than halving the mid-1960s to mid-1970s differential. This issue aside, Figures 3 and 4 show, between the mid-1960s and mid-1970s, smallish declines in life table deaths at all ages below 30 for males and most below 45 for females. A female exception is the age group 15–19, where a rise in the probability of dying (Fig. 5) likely reflects life table error. The ‘saw-tooth’ character of the lower graphs in Figure 5 challenges the detailed accuracy of SPC life tables.

Increments in life table deaths to balance declines at younger ages between the mid-1960s and mid-1970s are concentrated at older ages (Figs 3,4), but with some idiosyncrasies of dubious authenticity also evident (males aged 50–54; females aged 45–49 and 70–74). There is an indication of improved female mortality at reproductive ages, but a gender difference pointing to improved maternal mortality *per se* is less clear-cut than in the upper-right graphs of Figures 3–5. Ministry of Public Health (2005: fig. 5.1) data do, however, claim a steep decline in maternal mortality over this period.

Graphs for the mid-1970s to mid-1980s in Figures 3 and 4 show life table deaths falling at the youngest ages and in early middle age (30–54 years for males; 30–49 years for females), and rising at older ages. Males exhibit a major reduction in infant deaths and a large one in early childhood deaths. The female decline in infant deaths is smaller, but it is larger in early childhood and there is a larger decline at ages five to nine. Whether these differences mean much is unclear; Figure 5 shows similar trends, of differing magnitudes, for the sexes across these three age groups. From the mid-1970s UNICEF funded a concerted drive to train traditional birth attendants (Porapakkham, 1986). Births delivered by trained personnel consequently rose from 15.1% in 1976 to 26.9% in 1979 and 50.3% in 1982 (Porapakkham, 1986: table 79) before reaching 69% by 1987 and 99% by 2000 (World Bank, 2010).

Concerning childhood mortality, 1976–1986 embraced the Fourth and Fifth Five-year Plans, within which maternal and child health (MCH) initiatives were explicit policy objectives. The Fourth Plan (1976–1981) was broadly focused on ‘betterment of the health of the rural population’ and had more specific goals of ‘providing (an) organized programme of MCH services’ and ‘improv(ing) nutritional status’ (Porapakkham, 1986: 100). It saw the 1978 launch of the Rural Primary Health Care Expansion Project, which the Fifth Plan (1981–1986) sought to build on. It aimed to further improve nutrition through educating mothers, providing school lunches in 5000 impoverished areas, and other initiatives, and to expand immunisation of children against communicable diseases (Porapakkham, 1986). Preschoolers with ‘normal’ nutrition rose from 47% to 79% during 1982–1989 (Nitayarumphong, 1990). BCG vaccination coverage of infants, 54% in 1979, reached 90% by 1986, while DPT and oral polio vaccination rose from 21% to 74% and from 34% to 72% of infants during 1982–1986 (Ministry of Public Health, 2005: table 5.8). Trained medical personnel and health infrastructure also expanded. During 1979–1987 the population per doctor ratio outside Bangkok fell from 16 133 to 8872, that per trained nurse from 5286 to 2681, and that per inpatient bed from 881 to 723 (Ministry of Public Health, 2005: tables 6.5, 6.23 and 6.30). Community hospitals doubled from 254 in 1977 to 557 in 1987, and health centres increased from 4088 in 1979 to 6992 in 1987 (Ministry of Public Health, 2005: tables 6.33 and 6.37).

This growth in rural health personnel and infrastructure may also largely explain the declines in life table deaths in early middle age between the mid-1970s and mid-1980s (Figs 3,4). During this decade primary health care was implemented nationwide. Lessons learned from earlier experiments were applied, with 98.4% of villages covered by 62 000 VHVs and 588 000 VHCs by 1989 (Nitayarumphong, 1990), and access to basic health care, safe water, and adequate sanitation expanded. Households with ‘safe’ drinking water rose from 13.5% to 65.9%; those with ‘sanitary’ latrines rose from 33.9% to 47.1% (McGuire, 2003a: table 5). These trends and better access to medical treatment reduced the incidence of once-fatal conditions and the mortality from cases that did arise. The comparison of mid-1980s with mid-1970s _n_q_x_-values (Fig. 5) shows, with minor exceptions, distinctly lower probabilities of death at ages below 55 (males) and 50 (females), and essentially unchanged probabilities at older ages. An analysis of Thai cause of death data for the 1970s and early 1980s (Porapakkham and Prasartkul, 1986) documents a continued transition from infectious to degenerative causes, and marked declines in under-five mortality from upper respiratory infections, diarrhoeal disease, and pneumonia. It also shows reduced adult infectious disease mortality benefitting early middle more than older age groups.

### Mortality trends mid-1980s to present

**Figure 6:**
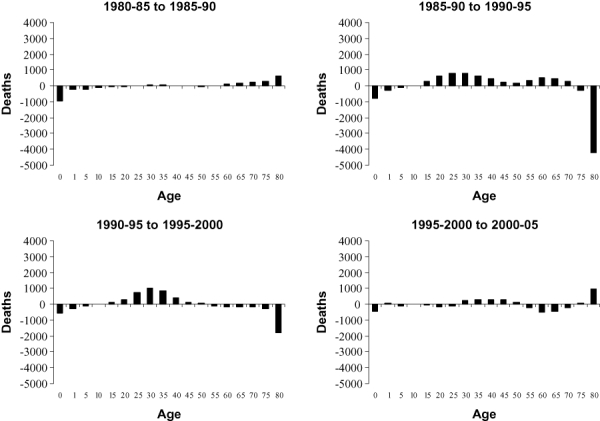
Changes in life table deaths between successive male life tables, 1980–1985 to 2000–2005 (radix = 100 000)

**Figure 7:**
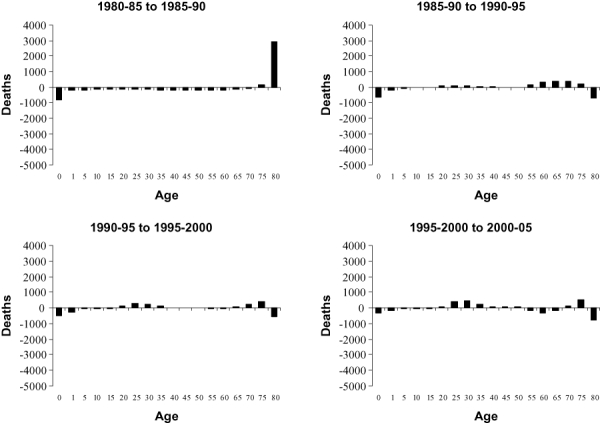
Changes in life table deaths between successive female life tables, 1980–1985 to 2000–2005 (radix = 100 000)

Trends in life table deaths by age and sex across successive quinquennia (not decades) between 1980–1985 and 2000–2005 are shown in Figures 6 and 7. These graphs are based on the UN life tables incorporating AIDS mortality. Data for 2000–2005 to 2005–2010 are not shown because they do not factor in the impact of ART. Those for 1995–2000 to 2000–2005 are also impacted by this problem, but more modestly, as the ‘extraordinary expansion of the Thai ART programme’ occurred during the latter period (World Health Organization and (Thai) Ministry of Public Health, 2004: 8).

The SPC-based lower-right graphs in Figures 3 and 4 overlap with upper graphs in Figures 6 and 7. The former show further reductions in infant mortality and significant improvements in adult mortality, this time concentrated in late middle age and early old age. They thus form an interesting sequel to SPC-based graphs for the previous decade, suggesting improvement in health infrastructure and the health system first boosted survival prospects in early middle age, then at older adult ages. Growth in health personnel and infrastructure certainly continued, as did improvement in access to safe drinking water and sanitation. During 1987–1995, populations per doctor, trained nurse and inpatient bed outside Bangkok fell from 8872 to 6243, from 2681 to 1407, and from 723 to 576, respectively (Ministry of Public Health, 2005: tables 6.5, 6.23 and 6.30). Community hospitals increased from 557 in 1987 to 688 in 1995, and health centres increased from 6992 in 1987 to 8842 in 1996 (Ministry of Public Health, 2005: tables 6.33 and 6.37). During 1985–1995, households with safe drinking water rose from 65.9% to 92.2%, and those with sanitary latrines increased from 47.1% to 96.1% (McGuire, 2003a: table 5). Outpatient visits increased from 11.9 million in 1977 to 30.9 million in 1985 and 73.1 million in 1995 (Ministry of Public Health, 2005: table 6.47).

Notably, however, the SPC-based lower-right graph in Figure 3 carries no imprint of the sharp rise in male traffic fatalities between the mid-1980s and mid-1990s. There is, by contrast, a clear imprint in the UN-based upper-right graph of Figure 6. Besides showing evidence of a continuous reduction in infant and child mortality apparent across Figures 6 and 7, this graph exhibits sharply deteriorating survivorship across ages 15–44 and 55–74, compensated by substantially fewer life table deaths at the oldest ages. The pattern is perhaps indicative of younger victims being vehicle drivers/riders/passengers and older ones often pedestrians, a speculation supported by evidence in the corresponding Figure 7 graph of females also being affected at older ages. It makes sense that pedestrian victims were not sharply gender differentiated. The contrasting patterns in the lower-right graphs of Figures 3 and 4 and the upper-right graphs of Figures 6 and 7 are puzzling, though. Both have a degree of plausibility, and perhaps the reality across the late 1980s and early 1990s was a combination of the two. The pattern evident in SPC data is not, however, also evident in the earliest UN data (between 1980–1985 and 1985–1990).

**Figure 8:**
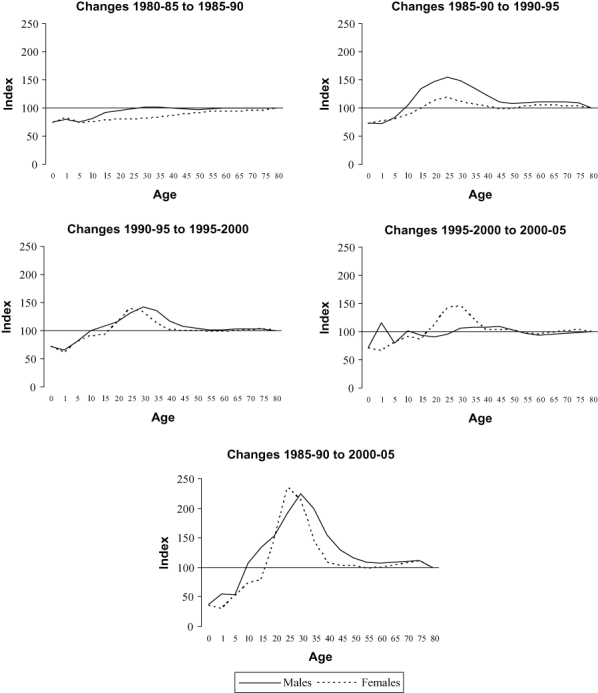
Changes in age-specific probabilities of dying between successive life tables by sex, 1980–1985 to 2000–2005

If AIDS deaths did not occur in large numbers until the mid-1990s, a decade after the first AIDS case was reported, graphs capturing AIDS mortality are the lower two in Figures 6 and 7, and corresponding graphs in Figure 8. The 1990–1995 to 1995–2000 graph in Figure 6 shows marked increases in male life table deaths at ages 20–44, peaking for the age group 30–34. Corresponding proportionate increments in probabilities of dying at those ages are traced in Figure 8 and look little different to those for females until it is remembered that they are calculated on bases (index values of 100) that incorporate the earlier large rises in young adult male road accident mortality. Probabilities of men dying aged 20–24, 25–29, and 30–34 in 1990–1995 were well over three times female equivalents, and were also 2.7 and 2.3 times higher at ages 35–39 and 40–44. There were further, more modest increments in male life table deaths at ages 30–49 between the late 1990s and early 2000s (Fig. 6), after which young adult male mortality probably improved under the impact of behaviour modification (greatly reduced patronage of sex workers – see Celantano *et al*., 1998; Ngamprapasom, 2001) and ART.

The imprint of AIDS on female mortality in Figure 7 is different. More modest increases in life table deaths are evident in the late 1990s because it was mainly sex workers who succumbed early in the epidemic. Larger increments in young adult life table deaths then occurred as male clients infected their wives and other non-prostitute partners.

In Figure 8 the lower graph summarises increments in adult mortality between the late 1980s and early 2000s – across the 15-year period embracing the upsurge in road fatalities and the main mortality impact of AIDS. Despite the Asian economic crisis it was a period of major improvement in infant and childhood mortality for both sexes, and of some improvement for females aged 10–19 as well. The two peaks occur at roughly similar levels that represent a doubling and more of mortality but are offset five years in keeping with sex workers being on average younger than their clients. The male ‘mountain’ is also broader based, reflecting clients' wider age range and, not least at ages 15–19 where little AIDS mortality (as distinct from infection) will have occurred, the contribution made by road deaths. The narrower female ‘mountain’ is to a greater extent AIDS generated, but again it must be appreciated that index values of 100 set different starting points for the sexes, male probabilities of dying aged 20–24 through 35–39 in 1985–1990 being 2.5, 2.7, 2.5 and 2.1 times those for females. It follows that the twin shocks of increased road deaths and AIDS actually failed to raise female young adult mortality to levels already prevailing among males before the shocks occurred. On the other hand, at ages 25–29 and 30–34, male probabilities of dying in 2000–2005 were five-and-a-half times pre-shock (1985–1990) female levels.

Hill *et al*. (2007) do not provide _5_q_x_-values^6^ from Thai life tables they construct for the 1980s and 1990s to facilitate comparison of increases in mortality risk for five-year age groups with UN increases. They rely on _45_q_15_ (the probability of dying between ages 15 and 60) as a summary index of ‘adult’ mortality. It rose for males in their life tables by 35% between the 1980s and 1990s, and fell for females by 1%. If compared with the 1985–1990 and 1995–2000 UN life tables, the male rise is 32%, and there is a more modest female rise of 6–7%. Extending the comparison period to 2000–2005 these increases reach 34% and almost 15%, respectively. The gender difference in the timing of these trends is consistent with men, aside from being far more affected by rising traffic mortality early in this period, having also been at the forefront of the AIDS epidemic. They had then begun to modify their behaviour (e.g. by eschewing sexual initiation with sex workers) by the time the epidemic spread among females beyond sex workers to the wives and other partners of infected men.

## Discussion

This paper has sought to establish what light life tables from several sources can shed on Thailand's mortality transition. *Doubts over the quality of the data in the studied life tables do cast a shadow over the exercise, but despite this, useful broad features of a transition that increased longevity in Thailand by around 30 years emerge.

The major initial impetus to transition came from the mass campaigns of the 1950s, which by 1960 had rendered steps to accelerate economic growth ‘well-nigh imperative’ (Ayal, 1961: 157) because population growth had reached 3% per annum and fertility remained high. Central was the spraying of DDT to combat malaria. The period 1947–1960 saw a huge shift in mortality from younger to older ages, the greatest proportionate improvements occurring in early adulthood but, assuming something approximating the ‘West’ model infant mortality in 1947, the largest absolute reductions in deaths occurring in infancy and early childhood. Older people's survival prospects also improved, but so many more reached old age that *numbers* of life table deaths at those ages could not but increase.

The 1960s, when experiments to expand primary health care began, saw further improvement in survivorship limited to infancy, early childhood and females of reproductive age. Under the First and Second Development Plans over 1200 midwife centres, and 143 ‘first-class’ and 1270 ‘second-class’ health centres were built, and under the Second Plan water supplies were developed in 23 800 villages (Porapakkham, 1986). Mortality gains in the early 1970s were spread across childhood and the early adult ages, probably including a further reduction in maternal mortality. Then between the mid-1970s and mid-1980s further gains in infancy and early childhood accompanied distinct gains in early middle age. According to SPC data, these shifted to later middle age and early old age in the following decade (along with further gains in infant mortality), the 20-year process reflecting the development of a nationwide primary health-care system with significant village-level community participation and ongoing initiatives to improve rural health infrastructure and access to health professionals.

Beyond the mid-1980s came a horrendous upsurge in road accident mortality and HIV/AIDS. Despite continued improvement in infant and early childhood mortality, the latest UN life tables suggest the former sent male life expectancy into decline between the late 1980s and early 1990s, AIDS then greatly accentuating this trend before a highly effective response to the epidemic (followed by ART) turned things around. Both traffic accidents and AIDS affected females less severely; upward momentum in life expectancy slowed to a standstill, but decline may have been averted. There can be no disputing, however, that rising traffic mortality and HIV/AIDS stopped a previously relentless post-war upward march of Thai life expectancy in its tracks after the mid-1980s.

The major feature of Thailand's mortality transition has perhaps been obscured by the period-by-period analysis presented here – inexorable improvement in infant and early childhood mortality. Doubt attaches to its precise extent through the mass campaigns era, but it was substantial. Beyond 1960, robust indirect estimates provide greater certainty, and currently quoted figures (World Bank, 2010) have infant mortality falling from 103 per 1000 live births in 1960 through 74 in 1970, 46 in 1980, 26 in 1990 and 11 in 2000 to below 7 in 2007. Under 5 mortality dropped from 149 per 1000 live births in 1960 to exactly 7 in 2007, meaning that very few children nowadays die aged one to four.

It cannot be argued that early post-war mortality decline in Thailand stemmed from economic development, but it may have spurred development by raising population growth to a level that by the late 1950s was sounding alarm bells (Ayal, 1961; Thomlinson, 1971), a process that a decade later yielded the National Family Planning Program to drive the second half of the country's demographic transition. Falling infant and child mortality is the centrepiece of any mortality transition, but while McGuire (2010) allows that a decline from 57% living below the poverty line in 1960 to 27% in 1990 and 10% in 2002 was important to Thailand's infant mortality revolution, that revolution did not, as its continuation and acceleration through the Asian economic crisis attests, occur as a simple function of the ebb and flow of GDP per capita. The tempo of public service provision – initiatives in education, family planning, and safe water and sanitation – was crucial. Fertility decline under the post-1970 National Family Planning Program was ‘breathtaking’ (Hirschman and Guest, 1990: 149), especially when in 1990 the population remained 71% rural. But cultural stars were aligned: the relatively high status of women, the absence of strong son preference, and a religion that did not oppose contraception and preached individual control of destiny. Ambitious rural water and sanitation policies followed in the 1980s (the ‘Safe Water and Environmental Sanitation Decade’), then efforts to greatly expand secondary schooling, which had languished, in the 1990s. Improved provision of basic rural health services through the 1980s and 1990s also helped, including the nutrition initiatives of 1981–1986 and efforts to expand the health workforce. The quality of care was suboptimal, with rural doctors disproportionately recent graduates and volunteers having limited training, commitment, and supervision, but did improve over time. Perhaps the best testament to this from an infant mortality viewpoint is the 1987–2000 rise from 69% to 99% of births attended by trained personnel. McGuire (2010: 249) credits the ‘democratic opening’ facilitated by three years of civilian rule during 1973–1976 as important in expanding primary health care – it spawned the Rural Doctors Forum, an organisation of progressive health professionals that agitated for that expansion. But the Thai mortality transition should not be viewed as simply a product of economic development. Early on, as Western technologies were exploited, it was a catalyst to development through its impact on population growth. Its continuation then became a product of development, but broadly, not narrowly economically, conceived.

## Footnotes

1 *Tambon* are a third-tier administrative unit in Thailand, below provinces (*changwat*) and districts (*amphoe*).

2 Period life tables are cross-sectional. They summarise a population's mortality experience based on age-specific probabilities of dying recorded in a single year or short period (commonly three years). Most life tables are of this type. They address the hypothetical question ‘If this population was to experience, through life, the mortality conditions that prevailed at each age during year (or short period) y, what would its collective mortality experience be?’

3 Cohort life tables trace the mortality experience of birth cohorts. They are less common because, to fully construct, they require data for around 100 years – the time it takes a birth cohort to fully die out. By comparison, as each new year's (or short period's) data become available a new period life table can be constructed *in full*.

4 ‘Model’ life tables are period life tables constructed by demographers to approximate likely mortality conditions in populations lacking the data to construct actual life tables. There is a skill involved in selecting the optimal series of model life tables (e.g. the ‘West’ series) for a population, but having done that and estimated a broad parameter like expectation of life at birth, a *particular* model table in the series can be consulted for the associated age by age mortality detail.

5 Any life table begins with an arbitrary number of persons alive at birth, known as its ‘radix’. Typically this is 100 000, and the life table follows this population's mortality experience until it dies out. Thus, there are a constant 100 000 ‘life table deaths’ in any life table, whose distribution by age can be compared between life tables.

6 Probabilities of dying for five-year age groups.

* Note: Correction added on 27 April 2011 after first publication online on 3 April 2011 in *Asia Pacific Viewpoint* Volume 52, Number 1. The sentence should begin with “Doubts over the quality of the data in the studied life tables do cast a shadow over the exercise, …” The error has been corrected in this version of the article.
